# Proceedings of the Ophthalmological Congress of Brussels

**Published:** 1859-01

**Authors:** 


ART. II.—*Congrès D'Ophthalmologie de Bruxelles. Compte-Rendu publié au nom du Bureau*, par le DR. WARLOMONT, Membre de l'Académie Royale de Médecine de Belgique, Chevalier des Ordres de Léopold, (de Belgique,) de Francois I[er], (de Naples,) des Saints Maurice et Lazare, (de Sardaigne,) Redacteur en Chef des Annales d'Oculistique, Secrétaire-Général du Congrès. Session de 1857. Paris: Victor Masson, 1858.

*Proceedings of the Ophthalmological Congress of Brussels, published by order of the Congress*, by DR. WARLOMONT, Secretary-General, etc. etc. Session of 1857. Paris: Victor Masson, 1858. 8vo., pp. 492.

IT is unnecessary to repeat the history of the Congress of which the official record is now before us in a well-arranged and handsome octavo volume of nearly five hundred closely printed pages. Many of our readers will recollect the call of the Committee of Belgian Oculists, at whose invitation and under whose able management this Congress was assembled. One hundred and fifty-nine ophthalmologists, including many of the

---

highest standing, were brought together, and arranged in three separate sections, for the purpose of discussing and deciding on a series of questions which had been published several months beforehand in the programme of the committee of arrangement.

The authority of the leading members of those sections, and the ample opportunity afforded them for preparation for the elaborate discussion of the topics previously submitted to them, as well as the importance of the topics in themselves, give a significance and value to the decisions of the Congress which entitle them to more attention and a wider circulation than they will be likely to receive in this country. They are sufficiently interesting to warrant a much more extended and detailed review than we can give of the various debates and papers of which they may be regarded as the qualified result, and, in some measure, the epitome. The best that we can do is to consider briefly the most important of these decisions, as they were finally adopted by the Congress and published in the appendix to the *Compte-rendu.*

The first section was entirely occupied with purulent and granular ophthalmia, or *military* ophthalmia, as they prefer to name it. The discussions and written articles on the questions relating to this form of ophthalmic disease, very naturally take up a large portion of the volume. Granular conjunctivitis and its sequelæ are, unhappily, common enough in the United States to engage at least an equally paramount attention; but it is among the ordinary, and especially the immigrant population, and not the military, that they are usually encountered on this side of the Atlantic. We do not see that the labors of our congressional debaters have developed anything under this head that is actually new; but they furnish us with a voluminous and instructive array of facts, and some excellent views of pathological structure and practical hints on treatment.

In the first place, the mode of transmission of purulent ophthalmia being inquired into, it was agreed that the disease was conveyed—

"1st. *By contact,* that is, by the transportation either directly (immediate contact) or through the agency (mediate contact) of contaminated objects.

"2d. *By infection.* Most frequently the transmission of the ophthalmia is effected through the medium of air charged with contagious principles. Some physicians, however, are not convinced that objects placed in an infected region can become impregnated with miasms, retain them a certain time, and, under favoring circumstances, restore them to the air and thus establish new centres of contagion. Crowding and insufficient aeration favor the development of foci of infection, in the midst of which young subjects, unhabituated persons, and raw recruits acquire the germ of contagious ophthalmia. These foci of infection aggravate the disease when developed, and increase its duration."

The second query asks, "*What part, in the transmissibility of ophthalmia, is to be assigned to granulations, and what is the nature of these latter?*" The answer to this, proposed in the Circular, covers two pages, and presents an excellent summary sketch, as well of the different varieties of granulations viewed anatomically, as of the distinct stages of the vesicular, the only specific form, regarded practically in connection with the course and termination of the disease and with its therapeutic treatment.

They soon found, however, that a general agreement as to the nature of granulations was, as usual, impossible; although there was evidently no material difference of opinion in regard to the kind of treatment required for what all recognized in practice as granular ophthalmia. The section, therefore, very wisely saved its time for better uses, by substituting, for the formal definitions, a non-committal generality, which answers the purpose of a waiver to one part of the question, while it rather seems to beg the other. It says that "whatever palbebral granulations may be understood to be, their presence most frequently causes a work of inflammation in the conjunctiva, with muco-purulent secretion, from which arise emanations which constitute one of the causes of the development of the granulations called *military.*" This substitute was sustained by the Congress in general session, after some opposition and a lively debate, which showed that a strong necessity for definite views of the nature of the granulations, to be submitted to the treatment recommended, was felt by some of the prominent members, and that an equally strong indisposition to admit the infectious nature of the muco-purulent emanations was entertained by others.

The solution of the next inquiry (C) was more fortunate in being unanimously accepted by the section and by the general Congress. Question and answer are well worth quoting:—"*Is there one formula of treatment the superiority of which, in the treatment of military ophthalmia, has been sanctioned by experience?*"

"The army ophthalmia is not a morbid entity, always the same, invariable in its cause, its course, and its symptoms, and therefore susceptible of being combated by a single kind of medication, whatever may be the value of this latter and the number of its cures. The employment of the numerous agents extolled by turns, has had the effect, alike of demonstrating the insufficiency of each exclusive method and of giving rise to a mixed method as variable as the form, symptoms, and complication of the ophthalmia. This is the method most prevalent at present, besides being the only rational one and the most efficacious.

"This treatment, into the details of which we cannot enter, consists, (*a*) *in acute purulent ophthalmia:* of the antiphlogistic medication in its whole extent, of the local application of certain agents intended to modify the conjunctival inflammation, of detersive injections, and of excision of the chemosis; (*b*) *in palpebral granulations:* of the employment of caustic or resolvent agents, combined, if need be, with antiphlogistic, alterative or tonic, and renovating medication, according to circumstances. In the choice of local means, preference must be given to those which are capable of contending with the disease without involving the integrity of the tissues, since experience has only too well taught the disastrous consequences of disorganizing applications. We must also know how to avoid too much or too frequent reaction, these being apt to convert the organs of vision into centres of fluxion, and to create diseased tendencies in them. On the other hand, if it is true that the value of any medication resides, for the most part, in the manner of putting it in practice, and that the very different results obtained from it often depend upon the *modus faciendi*, it is of the highest importance that the surgeon have well-settled ideas upon everything relating to the choice of preparations, their doses, and the mode of application.

"Finally, whatever be the treatment determined on, a proper application of the laws of hygiene should be the indispensable complement to the therapeutic management."

The third question (D) asks, "*What are the best measures to take to prevent the appearance and oppose the spread of military ophthalmia?*"

The answer to this is given, as unanimously adopted, under four general heads, in the following words :—

"The prophylaxis of the ophthalmia of armies comprehends :—

"I. The cure of the soldiers diseased at the time.

"II. The salubrification, and, if needed, the disinfection of the lodgings, (barracks, prisons, hospitals, etc.,) and of articles used by soldiers.

"III. Measures tending to check the propagation and the aggravation of the ophthalmia.

"IV. Measures intended to prevent the return of the disease when it has been once extinguished."

Under the first of these four heads are given fourteen different rules, comprising a complete and very precise code of regulations for the therapeutic and hygienic management of bodies of men affected with granulations. These rules being adapted to the requirements of the military hospitals and stations of continental Europe, are of less interest to American practitioners generally. They afford many hints, however, to those of us who are called upon to deal with the newly-landed denizens of our immigrant ships, or the occupants of our crowded railroad shanties, our mines, and all the holes and corners of our larger cities. The hospitals, alms-houses, and prisons, as well as the dispensaries of this country, furnish armies of granulation cases which are abundantly large enough to occupy us, without the additional burden of a swarm of military sufferers under the same crippling misfortune. The precepts adopted by the Congress in relation to the disease as observed among the soldiers, will apply, for the most part, equally well to the same affection as it prevails among the poorer classes generally, the chief difference being decidedly in favor of the soldier, on account of the discipline to which he is subjected, and the greater ease and certainty with which his treatment is controlled, as well as the general superiority of his quarters and of the diet within his reach.

Under the third head we find the following admonitions :—

"Military surgeons have become aware that in the double relation of treatment and prognosis an essential distinction must be made between *non-vascular* granulations and *vascular* granulations with blenorrhœa. It is known, on the other hand, that cases of army ophthalmia presenting, primitively, grave symptoms, are at present very rare: so it is at least in Belgium. The disease there is very mild at the outset and amenable to a simple and generally brief treatment. The obstinacy of the mischief and the accompanying dangers, arise only at a later period, when the inflammation has invested the conjunctiva, and this membrane is secreting a muco-purulent matter. Hence, the practitioner cannot exert himself too much in allaying the evil at the beginning, and in removing all influences capable of increasing its activity.

"Among these causes may be designated particularly :—

"(*a*) All those which tend to bring on a congestive or irritative condition of the visual organs or to occasion an inflammation of them; a faulty dress compressing the neck and scalp; prolonged exercises in warm weather; exposure to an atmosphere loaded with dust or smoke; the action of a strong light reflected from a white surface; a want of equilibrium between the cutaneous and mucous systems; checks to perspiration, and too irritating or caustic applications to the conjunctiva; and, finally, gonorrhœa, which has a most disastrous influence on soldiers affected with granulations.

"(*b*) The causes which disorder the nutritive functions, and thence exert an aggravating influence on the progress of the disease; abuse of alcoholic liquors; an unwholesome, indigestible, or insufficient diet; lodging in an atmosphere habitually cold, damp or vitiated.

"(*c*) Finally, overcrowding, [l'encombrement,] the cause to which the persistence of the evil in armies must, to a great extent, be attributed."

From these considerations certain valuable hygienic rules are deduced, and given at full length in nine additional and separate precepts, which we have not room to quote. Like the others previously mentioned, although proposed for military bodies, they are, in a great measure, applicable to other classes of granular patients, in the United States as well as in Europe.

Passing over the consideration of certain prophylactic measures, under the fourth head, recommended for the purpose of permanently eradicating and excluding granular ophthalmia from the military establishments, we proceed to the operations of the second section of the Congress.

The deliberations of this section were devoted to three very important questions in the recent progress of ophthalmology, and are so instructive and full of interest that we should be glad to have the space to occupy with the various practical points and reasonings, as well as facts, presented in the course of the discussions.

The first of these questions asks, "*What is the influence of the discovery of the ophthalmoscope on the diagnosis and treatment of diseases of the eye?*" There was no difference of opinion as to the advantage gained by this new and admirable means of exploration.

The answer of the committee was adopted, to the following effect:—

> "A crowd of affections of the refracting media and of the deep-seated membranes of the eye, hitherto difficult if not impossible to diagnosticate, are now recognized with the greatest precision by means of the ophthalmoscope. Such are: Incipient opacities of the crystalline humor; pathological alterations of the vitreous body; of the retina, and of the papilla of the optic nerve.
>
> "All rational curative indications resting evidently on the perfection of means of diagnosis, the ophthalmoscope, in giving a sure basis to these indications, has impressed upon the therapeutics of diseases of the interior of the eye a certainty and a precision which they did not possess before the introduction of its employment in science."

In adopting unanimously the solution, of which we have given above a literal translation, the whole section warmly applauded the ophthalmoscope as one of the finest inventions of our epoch, doing for the eye, as was aptly said, what the stethoscope has done for the chest. The speakers of the section, however, recognized the necessity of avoiding exclusive dependence on the physical exploration; functional examination being demanded to complete the anatomical data furnished by the ophthalmoscope. Even in the cases in which the instrument fails and the functional disturbances are alone available in the examination, among which cerebral amaurosis is the one most frequently encountered, the ophthalmoscope has its practical use, in affording material aid, at least in the means of diagnosis by exclusion, through its negative evidence as to the absence of all the affections which are accompanied with positive signs. The report of M. Crocq, (secretary of the second section,) to the Congress in general session, from which we quote these notes of the discussion, refers also to the suggestion of a member of the section, (M. Von Graeffe,) that in these cases of cerebral amaurosis the ophthalmoscope furnished even a positive fact of great importance in prognosis, in enabling us to determine whether or not there is any tendency to degeneration or atrophy of the retina. When this does not exist, the retina presents its normal aspect; it is pellucid with the usual vascular development. When it is present, the retina becomes opake, white, and its vessels incline to disappear; in the one case the prognosis being evidently favorable, and by no means so in the second.

Among the functional signs, the one which was the subject of particular discussion, as probably the most important to be noted in connection with ophthalmoscopic developments, was the condition of the phosphenes, to which M. Serre (d'Uzes) has called attention for some years past. The view, which was urged at some length, by M. Serre himself, in the session of the section, and subsequently, with still greater force and brilliancy, in the general session, advocated the utility and certainty of the phosphene test, not as the substitute for that afforded by the ophthalmoscope, but as an important coadjutor where the latter can be used, and as a conclusive means of examination in cases of anterior opacity in which the ophthalmoscope is powerless.

We are unable to pursue the discussion of ophthalmic exploration reported in the *Compte-rendu*, important and interesting as the proceedings of the Congress prove it to have been. The whole subject, as exhibited in the *Compte-rendu* alone, demands a full consideration by itself, rather than the cursory allusion to which we are necessarily confined.

The remarks of MM. Heyman, Von Graeffe, Sichel, Jaeger, and Donders, on the changes of the crystalline lens and capsule, as observed with the ophthalmoscope and without it, and incidentally on the seat and mode of extension of cataractous striæ, as well as of other opacities of the lenticular body and its envelope, are all extremely interesting. Still more interesting are the observations of these and other distinguished members of the Congress, in relation to diseases of the vitreous humor, the choroid coat and the retina.

Question third of the programme inquires, "*What are the agents which concur in or preside over the accommodation of the eye?*"

The solution of the committee was adopted by the section, after full discussion, and accepted by the Congress without alteration.

It is sufficiently compact to enable us to present it without abbreviation:—

"Facts which throw some light upon this curious phenomenon are established in science. Our knowledge of these is chiefly due to the works of Helmholz.

"When the eye adapts itself to the vision of near objects:—

"1. The pupil contracts.

"2. The pupillary margin of the iris projects forward.

"3. The peripheral portion of this membrane inclines backward.

"4. The anterior face of the crystalline increases in convexity, and the central portion of this face advances toward the cornea.

"5. There is increased thickness of the crystalline, with diminished radius of its circumference and thinning of its borders.

"In the vision of distant objects:—

"1. The pupil dilates.

"2. The pupillary margin of the iris moves backward and its peripheral portion forward.

"3. The convexity of the anterior face of the crystalline diminishes and that face retires from the cornea.

"4. The radius of the circumference of the crystalline lens increases, and its central portion lessens in thickness.

"These divers changes may be explained by the modifications of curvature of the two faces of the crystalline; but, above all, by those of its anterior face, produced by the contraction or relaxation of the fibres of the iris and the corresponding movements of the ciliary muscle. These muscles, then, may be considered as the agents which preside over the accommodation or adaptation of the eye."

The fourth interrogatory of the programme comprises the three following propositions:—

"A.—*Does the present state of ophthalmological science authorize the admission of specific ophthalmias? In the affirmative, what is to be understood by this denomination, and to how many species of ophthalmia is it applicable?*

"B.—*Is the specificness of these affections recognizable by anatomical and physiological characters of the ocular globe?*

"C.—*Can a radical cure be obtained by simple topical applications, or does it always require the intervention of general treatment?*"

The answer, as modified by the section, after considerable discussion, and approved by the Congress, is given in the following three propositions:—

"1. It is demonstrated by statistics that a great number of cases of diseases of the eyes have their only, or at least predisposing cause, in the condition of the subject.

"The same anatomical lesions may, however, with few exceptions, develop themselves, when the diathesis, which is often the cause of it, does not exist.

"2. This being stated, general causes must be studied as much as possible in each case; if the clinical examination positively demonstrates their existence, we can, we ought indeed, to accept the etiological principle in the denomination of the disease. This practice will certainly exercise an advantageous influence on the progress of nosology and therapeutics; but it cannot be allowed to generalize the etiological nomenclature; it should be reserved exclusively for the cases in which a sufficiently evident relation is manifested between the diathesis and the anatomical form of the disease.

"3. The constitution and the causes derived from it, must be constantly taken into the account; it is possible that in thus proceeding we may be led by a more exact observation to find some differences among the anatomical lesions of diseases reputed similar but observed in different conditions of the constitution."

The most striking topic in the discussion of the question of specific ophthalmias, was that of glaucoma and its treatment according to the recent views of the Berlin professor, M. Von Graeffe. His notions of the pathology of acute and chronic glaucoma, and of the method and rationale of the treatment by incision of the iris, were zealously and brilliantly maintained by their author in the proceedings of the section, and a second time in the general meeting. This second demonstration was given by special request, and was received with decided acclamations of applause by the assembly. It is certainly an admirable history of the subject, in theory and practice; and proves the enterprise of the operator, whatever may be thought of his experience and philosophy.

The third section of the Congress was occupied with the last three questions of the programme, Nos. V., VI., and VII., as follows:—

"V. *Has experience established that certain forms of cataract can be cured without operation? In the affirmative, what are these forms and what are the means which may take the place of surgical means?*

"VI. *Of what utility is palpebral occlusion in the treatment of diseases of the eye? What are the affections of the eye which demand its employment, and what is the best mode of practicing it?*

"VII. A. *Would the existence of special establishments for the treatment of occular diseases be useful?* B. *Affirmatively, what conditions ought they to realize?*"

To the first of these questions we have the following answer, adopted without hesitation. It was endorsed with a very strong demonstration against the charlatanism which has ever tampered with the delusion of the curability of cataract without operation, as one of its most cherished opportunities in the miserable work of victimizing the unfortunate:—

"If by the word *cataract* is understood the *spontaneous* opacity (or opacity supervening under the influence of causes whose action is still unknown) which is produced more or less rapidly, in the substance of the crystalline lens, we may reply without hesitation: NO, there is not any authentic fact on record in the annals of science to demonstrate that a cataract has ever retrograded or been arrested in its march under the influence of any medical treatment whatever.

"If the denomination of *cataract* be applied to the opacities of the crystalline which are the sequel of traumatic lesions, there do exist some facts demonstrating that an energetic antiphlogistic treatment has succeeded in arresting the development of these opacities, in limiting their extent, or even in causing their diminution when already formed.

"If, finally, by the word *cataract* is meant opacities of the capsule, which, in the immense majority of cases, not to say in all, are but secondary depots from inflammations of the iris or of the membrane of the aqueous humor, experience has shown that the disappearance of the opacity may often be obtained by the employment of a treatment adapted to these latter affections."

Question sixth refers to an expedient in treatment which has attracted much attention among ophthalmologists for some time past, especially among those who constituted the majority of the Brussels Congress. The discussion was an animated one and evidenced considerable difference of opinion as well as a great deal of special practical experience, all of which was reconciled in a brief decision, which is short enough to enable us to quote it in full:—

"Palpebral occlusion is intended to render the eyelids immovable; to withdraw the eyeball from the action of the air and of the foreign bodies therein suspended, and to hold it; to favor the action of remedies in prolonging their contact with the occulo-palpebral apparatus, and finally to admit of the maintenance at will of a fixed temperature.

"For these divers purposes, it may be useful in ulcers and perforations of the cornea; in protrusion of this membrane and hernia of the iris; in recent staphyloma; in ophthalmoptosis, and after certain operations performed upon the eye, such as of puncture and the operation for staphyloma; the operation for cataract; for artificial pupil, &c. Finally, much advantage also may be gained at times from the practice in ectropium, and in wounds with loss of substance of the external surface of the eyelids.

"The best mode of effecting it is that which responds most completely to the following conditions: To subject the eye to rest, and diminish as much as possible the rubbing between it and the lids; to confine it moderately, uniformly, and in such a manner as not to cause any pain; by no means to concentrate too much heat; to be renewable at will, without constraint or embarrassment, and to provide an outlet for the flow of healthy and unhealthy secretions.

"When the most complete immobility of the eye that is possible is desired, occlusion should be applied to both eyes, although but one may be affected."

The seventh and final question of the programme, relating exclusively to the utility and requirements of special hospitals for the eye, receives of course an affirmative answer. This emphatically demands in behalf of the well and the sick, the existence of special establishments for the treatment and the teaching of diseases of the eyes, as required by the interests of hygiene, therapeutics and science, and as a necessity of our epoch.

Then follow some details of construction and arrangement, advised as requisite in a well-ordered ophthalmic hospital, in addition to the general hygienic conditions proper to all hospitals.

"1. *General Exposure.*—One front to the east, another to the west.

"2. *Internal Distribution.*—(*a*) To be free from steps and other irregularities of the ground or floor against which blind patients might stumble.

"(*b*) The principal staircase should be wooden; should have equal, easy steps, straight landings, and railings on both sides.

"(*c*) Neutral, green, blue, and gray tints should form as much as possible the ground of the color of the walls and furniture.

"(*d*) Besides the wards or dormitories reserved for the lodgment of patients with non-contagious diseases, a few chambers, some for four beds, others for one or two, should be contrived, in which to place the patients operated on, and those having communicable diseases. These chambers should be furnished with green or dark-colored shutters, capable of regulating the amount of light.

"(*e*) Independently of out-door privies, inodorous water-closets should be placed on each floor for patients who are to be protected from exposure to the open air and injurious drafts.

"3. *Ventilation.*—As indispensable as in other hospitals, the ventilation ought to comply with the condition, so difficult to realize, of avoiding the creation of injurious drafts of air.

"Double doors, padded and listed, are very useful.

"4. *Grounds.*—The walks, yards, and gardens should be sheltered from the wind and the heat of the sun by thick trees.

"5. *Consultation and Operating Rooms.*—The chamber in which patients are examined or operated on, ought, as much as possible, to have but a single window, wide and facing north, in order to avoid the effects of light inevitable from any other disposition. Alongside of this chamber there should be a closet appropriated to ophthalmoscopic examinations."

In addition to the foregoing arrangements, amphitheatres, dissecting rooms, and other conveniencies are specified as needed for teaching purposes. It is also suggested that every medical community should have its own complete ophthalmic institute; and that the practitioners most familiar with ophthalmology should initiate the movement, each in his own country and place, in the confidence that they will find, in the municipal and general institutions, the support to which they shall prove themselves entitled.

Finally, a special chair of ophthalmology is recommended for each university or faculty of medicine.

In thus presenting a somewhat extended view of the formal decisions of the Congress, we have, as originally anticipated, exhausted our space so entirely as to be able only to refer to the many extremely interesting verbal and written communications from a large number of authoritative members and correspondents. We should have been glad, too, to have room for a few words upon the treatment of granulations, as suggested by the study of the debates and of the very elaborate and instructive memoirs, containing the results of a vast amount of intelligent and well-directed experience in different climates and countries as well as people. The subject of cataract also, and that of the ophthalmoscope, are treated, the one incidentally and the other formally, in such a manner and by such observers, as to suggest materials for separate reviews devoted to each topic, instead of the mere unsatisfactory mention with which we must content ourselves on this occasion.

The papers published in the Appendix occupy nearly three hundred closely printed pages, and are arranged in three different categories. Under the first of these (Memoirs on Military Ophthalmia) are twelve papers, some of them long, admirably arranged, and full of statistics on a very large scale, chiefly devoted to purulent and granular disease.

The next list (Memoirs on the State of Ophthalmology in different Countries) includes thirteen different articles, all interesting, and many of them full of statistical and historical as well as practical information; the first two (on Ophthalmology in America) being respectively from Drs. Gross and Littell, of Philadelphia.

The third division, under the caption of Various Communications, includes nine different contributions by as many authors; among which we notice an instructive and most complete paper, with marginal illustrations, on Ocular Prothesis or the Adaptation of Artificial Eyes, by M. Boissoneau, Sr., of Paris; one, also illustrated, on An Improved Method of Operating by Ligature for Staphyloma, by Dr. Borelli, of Turin; another, illustrated, by our countryman, Dr. Hays, of Philadelphia, describing his Knife-needle for the Division of Cataract; a note, by Dr. Delvigue, of Liege, on Ankyloblepharon, recommending as a remedial operation, the excision of a triangular flap of skin at the external commissure of the eye; a paper, by Dr. Kuchler, of Darmstadt, recommending a Mode of Treatment for Staphyloma and Chronic Inflammation of the Cornea; a note on a Simplification in the Operation for Cataract by Extraction, (dispensing with the incision of the capsule,) by Dr. Sperino, of Turin; a paper, by the same author, on Syphilitic Amaurosis, and on the Treatment, by Syphilization, of Syphilitic Ocular Affections; and a note on the same subject, by Dr. Boeck, of Christiania.

It is worthy of note that the only three American communications were sent from Philadelphia. Although interesting in themselves, they convey but little information as to the state of ophthalmology in this country. We regret, therefore, that the really able ophthalmotologists, in different portions of our Union, and especially in the larger cities, who are, undoubtedly, far more active than this meagre representation would imply, have neglected the opportunity to place themselves and their institutions in the proper light, by contributing to a general fund which has proved so rich a source of knowledge of what is being done for the advancement of their favorite branch in the greater part of the civilized world.

